# Genome-wide association, RNA-seq and iTRAQ analyses identify candidate genes controlling radicle length of wheat

**DOI:** 10.3389/fpls.2022.939544

**Published:** 2022-09-28

**Authors:** Fengdan Xu, Shulin Chen, Sumei Zhou, Chao Yue, Xiwen Yang, Xiang Zhang, Kehui Zhan, Dexian He

**Affiliations:** ^1^College of Agronomy of Henan Agricultural University/National Engineering Research Center for Wheat/Co-construction State Key Laboratory of Wheat and Maize Crop Science/Collaborative Innovation Center of Henan Grain Crops, Henan Agricultural University, Zhengzhou, China; ^2^Research Institute of Plant Nutrition and Resources and Environments, Henan Academy of Agricultural Sciences, Zhengzhou, China

**Keywords:** wheat (*Triticum aestivum* L.), radicle length, GWAS analysis, RNA-seq analysis, iTRAQ analysis

## Abstract

The radicle, present in the embryo of a seed, is the first root to emerge at germination, and its rapid growth is essential for establishment and survival of the seedling. However, there are few studies on the critical mechanisms underlying radicle and then radicle length in wheat seedlings, despite its importance as a food crop throughout the world. In the present study, 196 wheat accessions from the Huanghuai Wheat Region were screened to measure radicle length under 4 hydroponic culture environments over 3 years. Different expression genes and proteins (DEGs/DEPs) between accessions with extremely long [Yunong 949 (WRL1), Zhongyu 9,302 (WRL2)] and short roots [Yunong 201 (WRS1), Beijing 841 (WRS2)] were identified in 12 sets of root tissue samples by RNA-seq and iTRAQ (Isobaric tags for relative and absolute quantification). Phenotypic results showed that the elongation zone was significantly longer in root accessions with long roots compared to the short-rooted accessions. A genome-wide association study (GWAS) identified four stable chromosomal regions significantly associated with radicle length, among which 1A, 4A, and 7A chromosomes regions explained 7.17% to12.93% of the phenotypic variation. The omics studies identified the expression patterns of 24 DEGs/DEPs changed at both the transcriptional and protein levels. These DEGs/DEPs were mainly involved in carbon fixation in photosynthetic organisms, photosynthesis and phenylpropanoid biosynthesis pathways. *TraesCS1A02G104100* and *TraesCS2B02G519100* were involved in the biosynthesis of tricin-lignins in cell walls and may affect the extension of cell walls in the radicle elongation zone. A combination of GWAS and RNA-seq analyses revealed 19 DEGs with expression changes in the four accessions, among which, *TraesCS1A02G422700* (a cysteine-rich receptor-like protein kinase 6, CRK6) also showed upregulation in the comparison group by RNA-seq, iTRAQ, and qRT-PCR. BSMV-mediated gene silencing also showed that *TaCRK6* improves root development in wheat. Our data suggest that *TaCRK6* is a candidate gene regulating radicle length in wheat.

## Introduction

The plant root is essential to basic life, plant growth and development, and ground water and nutrient uptake and transport. The root tip is the most active tissue in the plant in terms of cell division and senses changes in the external environment, such as water, gravity, and light (Dietrich et al., 2017; Wang et al., 2020). Under drought stress conditions, nodal and lateral roots closer to the surface slow or stop growing, with the plant then relying on the primary root (seminal root) for absorption of water deep in the soil (Dietrich et al., 2017; Wang et al., 2020). The radicle is the first seminal root that breaks out of the seed coat during seed germination. The radicle develops from the promeristem, which differs from other secondary promeristem based on location, time and internal structure (Klepper, 1984).

There are numerous genetic markers associated with root growth and morphology in wheat. Canè et al. (2014) detected 48 quantitative trait loci (QTLs) for root morphology, three of which were associated with root length. These three QTLs were mapped to chromosomes 1A, 7A, and 7B, and explained 3.54%~14.23% of the phenotypic variation. Using a recombinant inbred line (RIL) population derived from Xiaoyan 54 × Beijing 411, Ren et al. (2012) found a QTL regulating root length from Xgwm 210 to Xbarc1138.2 on chromosomes 2B, explaining 68.0% of the phenotypic variation for maximum root length and 59.0% for total root length. Cao et al. (2014) mapped a QTL (*qTaLRO-B1*) to a region of 0.9 cM on chromosome 2BS using the near-isogenic lines (NILs) 178A and 178B. With the release of the wheat reference genome, many types of high-density SNP markers based on natural populations were analyzed by genome-wide association study (GWAS). For example, a GWAS on root traits in a natural population detected 1 QTL for seminal root length and 6 QTLs for nodal root length (Xu et al., 2021). Using GWAS and linkage analysis, Li et al. (2019a,b) detected three QTLs for total root length and root dry weight, distributed on chromosomes 5D and 6A. Beyer et al. (2019) detected four QTLs on chromosome 5B associated with seminal root length, and GWAS found 6 genes for root development near these QTLs.

Recent molecular techniques such as RNA-seq and iTRAQ can be used to find differentially expressed genes and proteins (DEGs/DEPs) between two comparison groups. Comparison of root growing in normal and drought conditions through RNA-seq has identified numerous DEGs involved in biological processes such as hormone biosynthesis and signaling, cell wall synthesis, and reactive oxygen species (ROS) metabolism (Zhu et al., 2008; Monika et al., 2018). Radical scavenging enzymes specifically regulate proteins in the cell wall and elongation region, such as ferritin proteins. There were significantly increased in roots of maize and soybean grown under low water potential (Zhu et al., 2006; Yamaguchi and Sharp, 2010). Using iTRAQ, Li et al. (2019a,b) determined that the differential distribution of ROS in the root tips of different genotypes may be related to the difference of primary root length. In addition, combination of GWAS and transcriptomic data can overcome the limitations of traditional gene mapping, and even predict the candidate gene. Combining QTL fine mapping, digital gene expression (DGE) and whole-genome re-sequencing (WGS), Hua et al. (2016) revealed a gene (NODULIN 26-LIKE INTRINSIC PROTEIN) regulating efficiency of boron uptake in the tetraploid rapeseed (Brassica napus). GWAS and transcriptomics have served as the basis for identifying 8 candidate genes for drought resistance (Zhang et al., 2015; Körber et al., 2016), 24 for disease resistance (Lu et al., 2017) and 14 for yield establishment of *Brassica napus* (Lu et al., 2017). Comparison of transcriptomic and proteomic data revealed that adventitious root formation correlates not only with hormone signal transduction but also with carbohydrate metabolism, energy metabolism, protein degradation and the activity of some transcription factors (TFs; Wang et al., 2019).

Previous research has scrambled for root development and responses in important food crops and many studies mainly focused on the total root system or maximum root length. However, there is no report on the molecular mechanisms that determining differences in wheat radicle length were rarely or even none. In view of this, 196 wheat accessions from the major wheat-growing region of China were grown under four hydroponic environments. The phenotypes were measured and their genotype was analyzed using the 660 K Wheat SNP Array to obtain GWAS results. Four accessions consistently showing extreme radicle length were further analyzed by RNA-seq and iTRAQ. This study used multiple techniques to clarify identify candidate genes responsible for the length of wheat radicle.

## Materials and methods

### Plant materials

A natural population of wheat was provided by the Collaborative Innovation Center of Henan Grain Crops, China, and consisted of 196 genetically diverse inbred lines. These accessions originated from nine provinces, namely Henan, Hebei, Shanxi, Shandong, Jiangsu, Shaanxi, Sichuan, and Anhui and Beijing (Supplementary Table S1).

### Growth conditions and measurement of radicle length

The nature population was planted in one indoor hydroponic environment and three outdoor hydroponic environments. The hydroponic method and nutrient solution were described by Xu et al. (2021). The indoor hydroponic culture experiment lasted 10 days in a greenhouse under a 16-h light, 20°C/8-h dark, 16°C photoperiod at 80% relative humidity with a light intensity of 180–200 μmol·m^−2^·s^−1^. Outdoor hydroponic culture experiments were performed from 20 October to 30 October 30 in 2016, 2017, and 2018 at Zhengzhou (34.7°N, 113.6°E), Henan, China. All wheat accessions were planted in a randomized block design with three replicates, two plants per replicate in each hydroponic experiment.

Four wheat accessions with extremely short/long radicle were identified [Yunong 949 (WRL1), Zhongyu 9,302 (WRL2), Yunong 201 (WRS1), Beijing 841 (WRS2)] and planted in a second indoor hydroponic experiment with three replicates, 100 plants per replicate. After 10 days, samples for microscopy and molecular analyses were taken.

For microscopy, seedlings were washed, and the root tips (1.5 cm) from five plants were excised and stored in FAA (acetic formaldehyde: alcohol: acetic acid: distilled water = 10:50:5:35) at 4°C until further analysis. Each root tip was dehydrated through an ethanol series (60%, 70%, 80%, 90%, and 100%; 0.5 h for each concentration). Then, the dehydrated samples were immersed in ethanol–xylene (1:1) and then 100% xylene for 2 h, embedded in paraffin, and cut into longitudinal sections of around 10 μm in thickness in a rotary microtome (prepared by the Wuhan Service Bio Technology, China). The sections were mounted on slides, deparaffinized, and stained with Safran in O-Fast Green staining for 5 min. The slides were viewed using a bright field scanning electron microscope (Pannoramic DESK, P-MIDI, P250, P1000, Hungary). The whole radicle from other seedlings were frozen immediately in liquid nitrogen and stored at −80°C for RNA-seq and iTRAQ.

### GWAS of radicle in wheat

All 196 accessions were genotyped using the Wheat 660 K SNP Array by Beijing Boao Crystal Code Biotechnology Co. Ltd.^1^ The genetic diversity, population structure, and LD analyses have been reported by Chen et al. (2020). The Array contained 390,136 SNP polymorphic markers with MAF (minor allele frequency) > 5, 15% missing markers were filtered from the SNPs used for GWAS.

GWAS was performed using six models [two generalized linear models (GLMs): quantile (Q) and principal component analysis (PCA); four mixed linear models (MLM): kinship (K), (PCA + K, and Q + K); a fixed and random model circulating probability unification (FarmCPU) combined with multi-locus mixed model (MMLM)] to obtain an optimized model by the quantile-quantile (Q-Q) plot of radicle length during germination in the indoor hydroponic environment. The best model was considered the one producing an actual −log_10_ (p) value closest to the expected −log_10_ (p) value (Supplementary Figure S1). Finally, GWAS was performed using a mixed linear model (MLM, Q + K) to analyze associations for each environment to radicle length in R (Liu et al., 2016). If the threshold was set at log_10_ (1/n) when the number of SNP markers was n, there was no significant SNPs associated with radicle length. To combine the GWAS results, the threshold of −log_10_ (p) was determined according to a uniform suggestive genome-wide significance threshold [−log_10_ (p) = 3.5; Beyer et al., 2019]. Manhattan and Q-Q plots were embellished using the CMplot package in R.^2^

### RNA isolation, illumina sequencing, and functional annotation of the transcriptome

To identify DEGs between accessions with extremely long and extremely short wheat radicle, total ribonucleic acid (RNA) was extracted from 4 extremely long and short wheat radicle using RNAiso Plus (TaKaRa, Otsu, Shiga, Japan) according to the manufacturer’s instructions. The RNA purification and library generation was according to Li et al. (2017). Library construction and sequencing were completed at the Beijing Genomics Institute (BGI; Shenzhen, China) using an IlluminaHiSeq™ 4,000. The primary sequencing data, or raw reads, were subjected to quality control (QC) according to Mortazavi et al. (2008). After QC, the raw reads were filtered into clean reads using “SOAPpnuke.”^3^ The clean reads were mapped to the Chinese Spring Wheat reference genome^4^ using “Bowtie2.”^5^ Gene expression levels were estimated by fragments per kilobase of transcript per million fragments mapped (FPKM) using a false discovery rate (FDR) of <0.001. A change of (|log2FC|) ≥ 1was set as the significance threshold for differentially expressed genes (DEGs; Benjamini and Yekutieli, 2001). The DEGs were annotated according to the Nr, Pfam, COG, Swiss-Prot, and KEGG Ortholog databases. KEGG Pathway^6^ and GO^7^ enrichment analyses were performed for target DEGs.

### Protein extraction, digestion, and iTRAQ labeling

The extremely long and short wheat radicle tissues from 4 accessions used for iTRAQ analysis were the same as those used for RNA sequencing (RNA-Seq). Three independent biological replicates were taken for each extreme cultivar for a total of 12 samples. Protein extraction and iTRAQ experiments were also completed at BGI (Shenzhen, China). Briefly, for each sample, 0.5 g tissue was ground into a fine powder in liquid nitrogen for protein extraction. Protein was extracted using acetone precipitation according to Wisniewski et al. (2009). Protein concentration was determined with a Bradford assay kit (BioRad, Hercules, CA, United States) using bovine serum albumin (BSA) as the internal standard. The protein quality was determined using 12% sodium dodecyl sulfate-polyacrylamide gel electrophoresis (SDS-PAGE). The supernatant from the acetone extraction was stored in aliquots at −80°C. Each sample (100 μg) was digested by Trypsin Gold (Promega, Madison, WI, United States) at 37°C (40:1, protein: trypsin), and desalted with a Strata XC18 column (Phenomenex, Torrance, CA, United States). Peptide samples were labeled using two iTRAQ 8-plex kits (AB Sciex Inc., MA, United States). One eight-plex iTRAQ set was used for eight samples with two biological replicates: the WRL1 samples were labeled with iTRAQ tags 113 or 117, WRL2 was labeled with tags 114 and 118, WRS1 was labeled with iTRAQ tags 115 and 119, and WRS2 was labeled with tags 116 and 121. Another eight-plex iTRAQ set was used for four samples (the third biological replicate) with iTRAQ tags 113, 114, 116, and 121. Data acquisition was performed using a Triple TOF 5600 System (SCIEX, Framingham, MA, United States) fitted with a Nanospray III source (SCIEX, Framingham, MA, United States). On-line separation was performed with an automated Easy-nLC1000 system coupled to a Q-Exactive mass spectrometer (Thermo Fisher Scientific, San Jose, United States). The high-sensitivity mode was set for survey scans.

Raw data files were transformed into MGF files using Proteo Wizard. The exported MGF files were searched using Mascot 2.3.02 (Matrix Science, Boston, MA, United States). NCBI-nr and Swiss Prot/UniProt database searches were performed for protein identification. In pair-wise comparisons of WRL1/WRS1, WRL1/WRS2, WRL2/WRS1, and WRL2/WRS2, DEPs were identified based on a fold change (FC) of ≥1.5 or FC ≤0.67 (*p* < 0.05, FDR ≤ 0.01). DEPs were searched against the GO, COG, and KEGG databases to classify and identify their potential functions. Classification and functional enrichment for the DEPs were determined by GO and KEGG pathway analyses (*p* < 0.05). Cellular locations of the DEPs were predicted through WoLF PSORT.^8^ The protein–protein interaction (PPI) of the identified DEP networks were analyzed using the online tool STRING 10.5.^9^

### Statistical analysis

The results were presented as mean, standard deviation (SD) and range of variation from three independent biological replicates. Graphical presentation of the data was generated using Microsoft Excel 2010. Single factor analysis of variance and multiple comparisons were performed to calculate statistical significance using SPSS 19, with a *p* value < 0.05 considered statistically significant with SSR. Venn Diagrams were generated using the online analysis tool Venny 2.1.^10^

### Integration of GWAS and transcriptome data

Based on the RNA-seq data, we calculated the ratios of upregulated and downregulated DEGs within stable genomic regions corresponding to significant SNPs from the GWAS. The DEGs that corresponded to radicle length genes detected in the GWAS were divided into several groups according to their unique expression patterns. The expression of candidate genes based on functional annotation was re-assessed by quantitative PCR analysis.

### Conjoint analysis of transcriptomic and proteomic data

The transcriptomic and proteomic data were compared by correlation analysis. The intersection was based on quantitative DEPs [(|log2FC|) ≥ 1] and DEGs (FC of ≥1.5 or FC ≤ 0.67) from the aspects of expression and functional enrichment (GO and KEGG). Pearson correlation tests were conducted using Spearman (Xiao et al., 2016) on each compared group. The DEGs/DEPs were divided into several groups based on whether transcript and protein levels showed the same trend, opposite trend, or no correlation. DEGs/DEPs that showed the same trend were subjected to classification and functional enrichment analyses. The expression of candidate genes based on conjoint analysis was re-assessed by quantitative PCR analysis.

### Quantitative RT-PCR

Based on the different omics analysis, the genes from stable regions determined in the GWAS or DEGs/DEPs that showed the same trend were selected for quantitative real-time (qRT-PCR) analyses for validation of the proteomics and transcriptomic data. Total RNA was used as the template for cDNA synthesis with reverse transcriptase (Takara Bio, Dalian, China) following the manufacturer’s instructions. Gene-specific primers were designed using Primer 5 software 46 (Supplementary Table S2). *β*-actin was used as an internal control gene. To validate the transcriptomic and proteomic profiles produced in this study, 4 genes from the GWAS-RNA-seq and 9 genes from the RNA-seq-iTRAQ combinations were randomly selected for qRT-PCR analysis.

The diluted cDNA samples (100 ng/ml) were subjected to qRT-PCR using the Dice Real-Time System III (Bio-Rad Laboratories, Inc., Hercules, CA, United States). The reactions were performed in a CFX96 Real-Time PCR Detection System (Bio-Rad Laboratories). The qRT-PCR program was as follows: 95°C for 30 s; 40 cycles of 95°C for 5 s, 60°C for 30 s and 72°C for 15 s; 95°C for 5 s and 55°C for 30 s and 72°C for 60 s. Relative gene expression levels were calculated according to the 2^−ΔΔCt^ method (Livak and Schmittgen, 2001).

### System of VIGS

Wheat cultivar Yunong 949 exhibited high sensitivity to barley stripe mosaic virus (BSMV) and was used to conduct VIGS. PCR amplification with gene-specific primers (Supplementary Table S2) generated 358-bp fragments for the candidate gene. Vector constructs, plasmids linearization, and *in vitro* transcripts were performed as previously described (Zhang et al., 2016; Kang et al., 2019). One hundred plants of wheat were infected with BSMV-PDS (positive control) or BSMV-candidate gene construct, with inoculation with wild type (WT, negative control) as a control in each experiment. A volume corresponding to 0.01 g viral RNA was rub-inoculated onto the second leaf of per silenced seedling at the 2–3 leaf stage (10 days after sowing). The root tissues (0.2 g) were collected from each treatment group 10 days post-inoculation for qRT-PCR to determine the efficiency of silencing of the candidate gene. The success rates of the plants (15 seedlings per biological replicate) inoculated with different BSMVs was recorded at 10 days post-inoculation, and this experiment was repeated three times for each BSMV.

## Results

### Phenotypic variations in wheat radicle length

**Figure 1:**
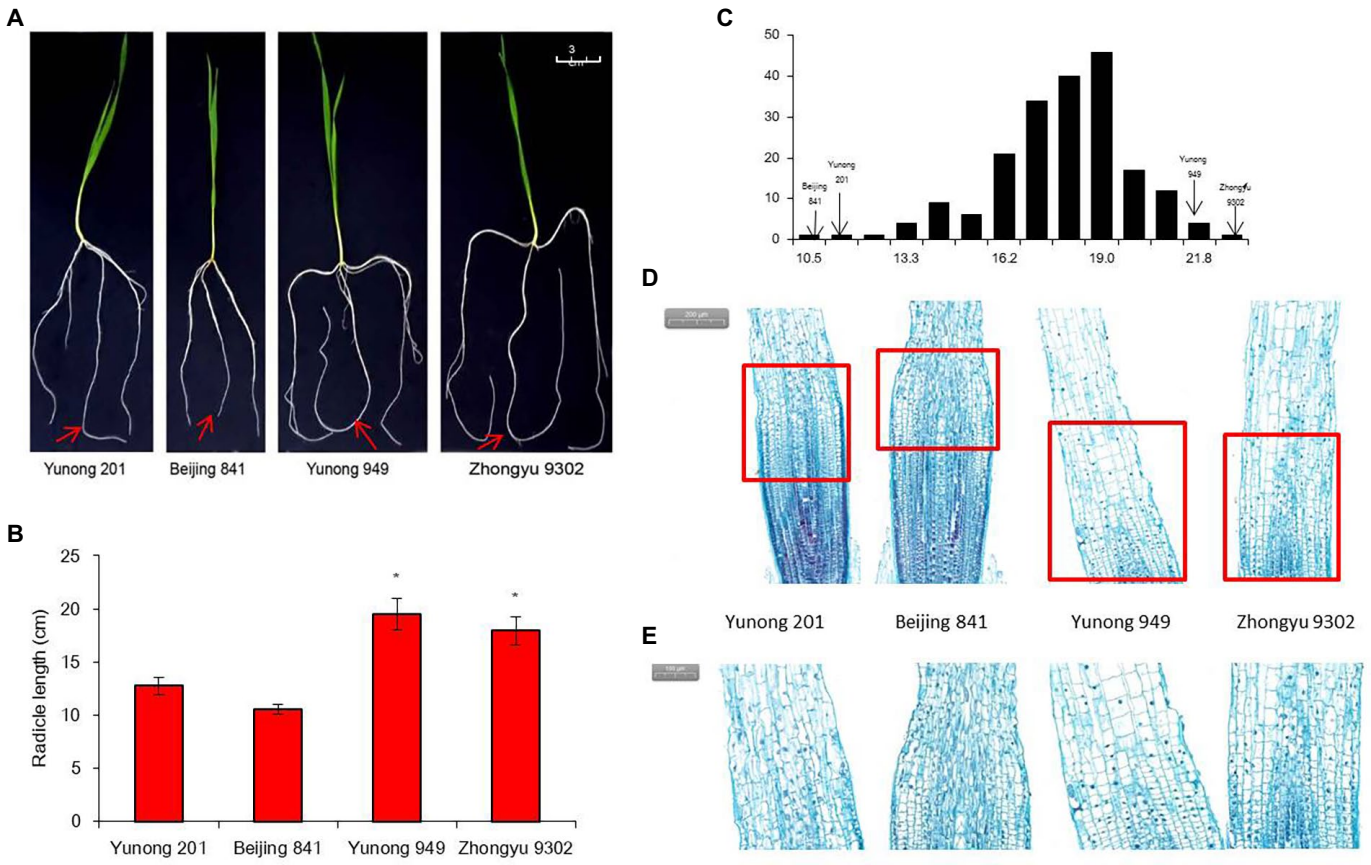
Radicle length after 10 days of hydroponic growth in different accessions. **(A)** Images of the radicle length. Red arrows indicate radicle; **(B)** Average radicle length difference; **(C)** Frequency distribution of the radicle length for 196 accessions in the four hydroponic environments; **(D)** Image of radicle tips in bright field microscopy with stain. Red frames indicate elongation zones; **(E)** Elongation zone in radicle tip. Significant differences are indicated as *p* < 0.05 (*).

The distribution frequency of the root length showed continuous variation and a normal distribution (Figure 1C). Among the 196 accessions, two accessions had significantly longer radicles, namely Yunong 949 (WRL1, 21.2 cm) and Zhongyu 9,302 (WRL2, 18.6 cm), while Yunong 201 (WRS1, 13.2 cm) and Beijing 841 (WRS2, 10.8 cm) had significantly shorter radicles (Figures 1A,B). Microscopy of the radicle tips revealed that, in the accessions with longer roots, the cells in the elongation zone were longer and more closely arranged than those in the short-root accessions (Figures 1D,E). What is more, elongation zone length and cell length of root tip between long-root accessions and short-root accessions reached a significant level (Table 1).

**Table 1:** Statistics radicle length and root-tip cells of extreme accessions after sowing 10 days.

Accession	Radicle length (cm)	Elongation zone length (mm)	Cell number of elongation zone (no.)	Cell length of elongation zone (μm)
Yunong 201	13.22b	1.08b	327.82a	48.33b
Beijing 841	10.83b	0.99b	305.44a	42.47b
Yunong 949	21.24a	3.23a	359.21a	59.82a
Zhongyu9302	18.64a	2.96a	364.53a	60.01a

Values followed by different letters in a column mean significant difference among treatments (*p* < 0.05).

### GWAS for radicle length

**Figure 2:**
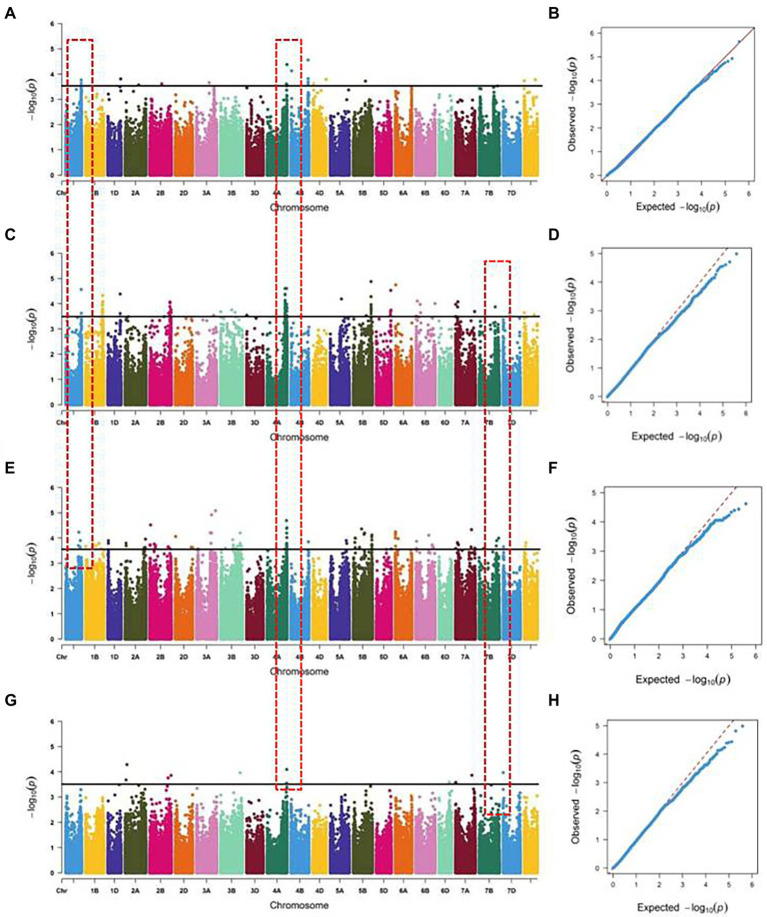
Quantile-quantile plots and Manhattan plots for radicle length across 196 accessions and SNP chromosome locations. **(A,B)** Wheat hydroponically grown in greenhouse; **(C,D)** Wheat hydroponically grown outdoor in 2017; **(E,F)** Wheat hydroponically grown outdoor in 2018; **(G,H)** Wheat hydroponically grown outdoor in 2019.

For a quality control check of the genotypic data, the wheat 660 K SNP Array identified 390,136 polymorphic SNPs with MAF > 5%. GWAS was performed between the SNPs and radicle length of 196 wheat accessions in four environments. Based on the optimal models, 85 SNPs were significantly associated with radicle length from different environments. The SNPs were distributed across all chromosomes except for 2D and 5A, with R^2^ values ranging from 7.02 to 14.04% (Figure 2; Supplementary Table S3). Thirty six SNPs were detected from two or more environments at similar physical intervals. Using 10 Mb of linkage disequilibrium (LD) decay distance as a confidence interval (Chen et al., 2020; Xu et al., 2021), a total of four stable regions were detected, located on chromosomes 1A, 4A, and 7A. The 719.89~728.34 and 731.34~741.59 Mb regions on chromosome 4A were detected in all four environments, with R^2^ values ranging from 7.02%–12.39%, which was not reported before. The 570.52~580.23 Mb region on chromosome 1A was detected in the indoor and two outdoor environments, with an R^2^ range of 7.86%–10.09%. The 7.59~10.20 Mb region on chromosome 7A was detected in the three outdoor environments, with an R^2^ value ranging from 7.86%–10.09%.

### RNA-seq analysis and identification of DEGs

**Figure 3:**
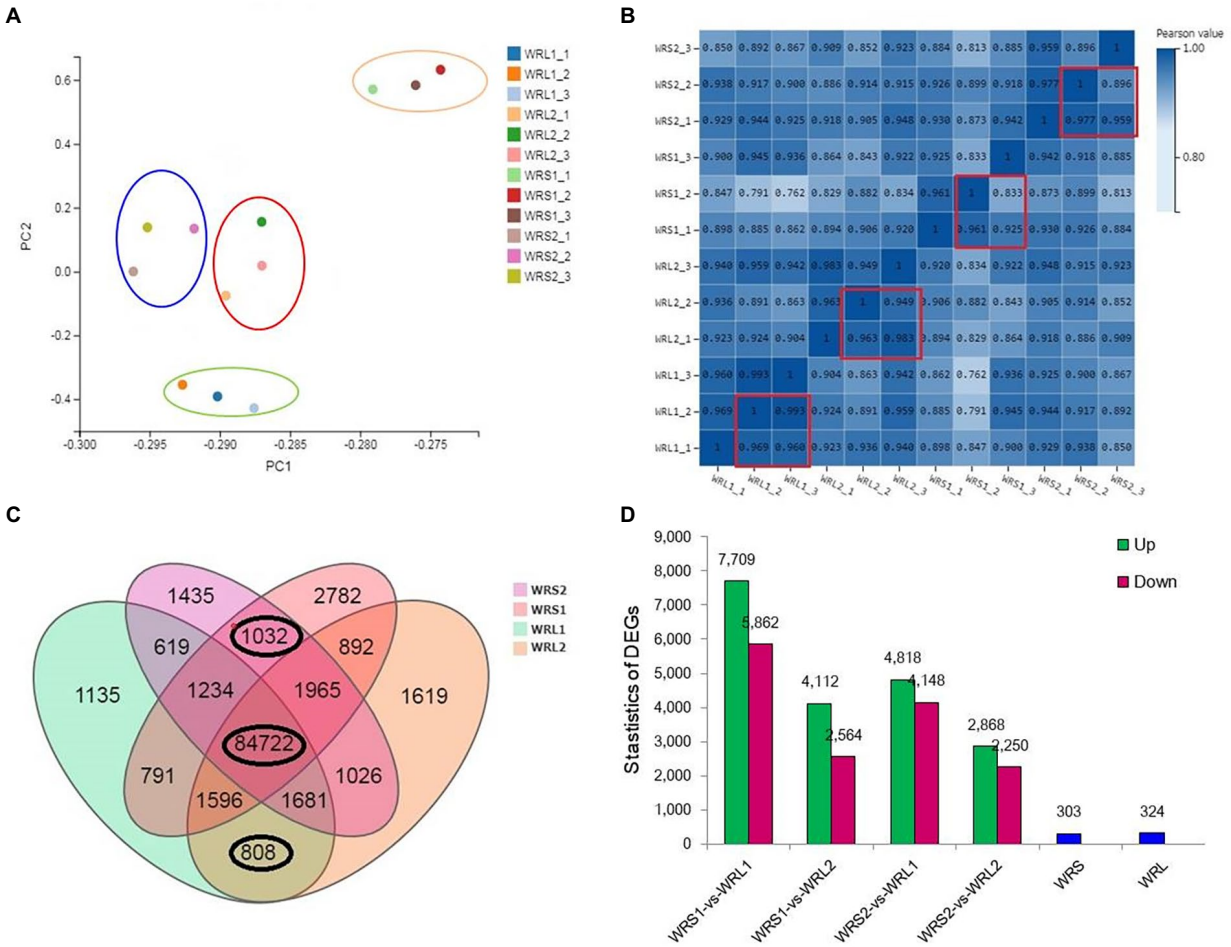
Transcriptome data of wheat accessions with long and short radicles. **(A,B)** Quality control of all samples; **(C)** Number of transcripts per accession; **(D)** Number of DEGs in pair-wise comparisons.

To identify genes potentially involved in radicle length, RNA-seq was performed on roots of 10-days-old plants of the two shortest and two longest accessions, with three repetitions. The principal component analysis (PCA) and Pearson correlation coefficients of all samples revealed good repeatability among the biological replicates, although the partitioning of WRL2 (red circle) and WRS2 (blue circle) was not obvious with higher coefficients (0.9; Figures 3A,B). In total, we obtained 156,590 genes/transcripts and 122.34 Gb clean reads from the 12 samples, with 10 Gb clean reads on average per sample and clean read ratio values over 90% for all samples (Supplementary Table S4). Among them, 84,722 genes were expressed in all four accessions (FPKM ≥ 0, FDR ≤ 0.001; Figure 3C). The criteria (fold change greater than 2, FPKM ≥ 0.1, FDR ≤ 0.001) used as the threshold to judge the significant differences in gene expression were stringent. There were 324 genes expressed specifically in WRL and 303 genes specific to WRS. Pair-wise comparisons identified 8,821 (WRS1/WRL1), 4,339 (WRS1/WRL2), 5,828 (WRS2/WRL1) and 3,326 (WRS2/WRL2) DEGs (Figure 3D; Supplementary Table S5), and 3,086 overlapping DEGs.

In the GO analysis, three main GO categories, biological processes (BPs), cellular components (CCs) and molecular functions (MFs), were divided in 15, 14, and 10 functional subcategories according to common DEGs of WRS/WRL (Supplementary Figure S2). The most enriched GO terms among those involved in the BPs were in cellular and metabolic processes, followed by response to stimulus and biological regulation. Among the CCs, cell, organelle part, membrane and membrane part were significantly enriched. Among the MFs, the top four terms involving the most DEGs were binding, catalytic activity, transporter activity and transcription regulator activity. To further determine which biological pathway significantly regulated the length of wheat radicle, a KEGG pathway enrichment analysis was performed. The KEGG analysis indicated that 122 pathways were enriched in DEGs (Supplementary Figure S3). These pathways were mainly related to metabolism (76), genetic information processing (19), cell processing (4), environmental information processing (4), environment adaptation (4), and organismal systems (2). The 21 KEGG pathways with remarkable enrichment of DEGs (*p-*value < 0.05) are listed in Supplementary Table S6. DEGs were mostly enriched in phenylpropanoid biosynthesis (128), followed by carbon metabolism, then photosynthesis-antenna proteins, glutathione metabolism, phenylalanine metabolism, and glyoxylate and dicarboxylate metabolism, among others. The genes that were specifically expressed in WRL were most enriched in plant-pathogen interaction, ubiquitin-mediated proteolysis, protein processing in endoplasmic reticulum, phagosome, and oxidative phosphorylation (Supplementary Figure S4A). The genes that were specifically expressed in WRS were most enriched in MAPK signaling pathway-plant, plant-pathogen interaction, plant hormone signal transduction, phenylpropanoid biosynthesis, phagosome and oxidative phosphorylation (Supplementary Figure S4B).

### iTRAQ analysis of DEPs

**Figure 4:**
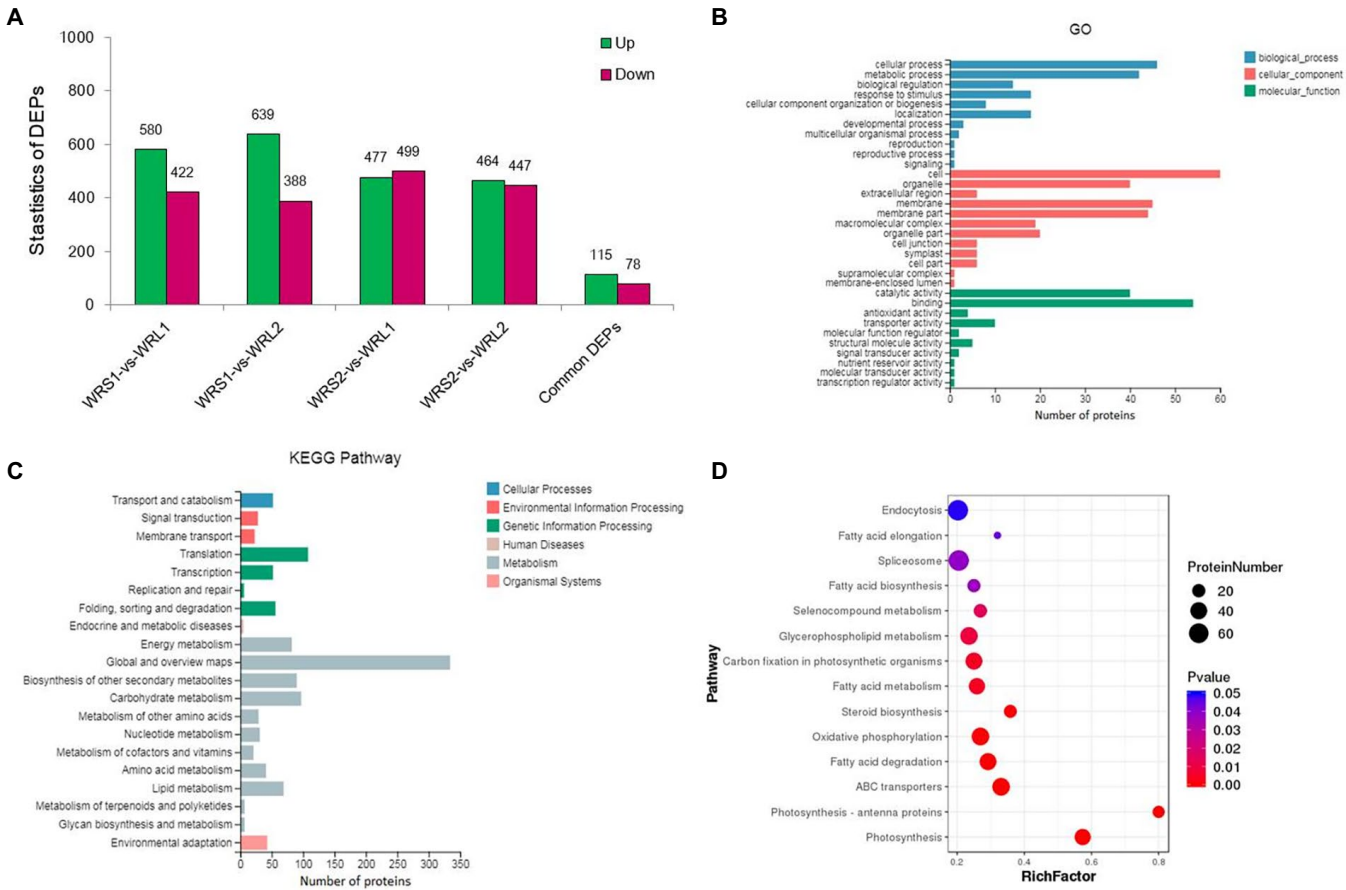
Statistics and annotation of the DEPs in short and long radicles of wheat. **(A)** Venn diagram of the number of proteins identified in 3 replicates; **(B)** Number of DEPs in pair-wise comparisons; **(C)** DEP-enriched GO annotations; **(D)** DEP-enriched KEGG annotations.

To identify DEPs linked to radicle length, the samples used for RNA-Seq (WRS1, WRS2, WRL1, and WRL2) were also used for proteomics analysis. We obtained 2,775,498 spectra from 12 samples by iTRAQ analysis, of which, 61,902 peptides were mapped, finally identifying 14,895 proteins (Figure 4A; Supplementary Table S7). The criteria used as the threshold to judge the significant differences in the protein expression were stringent (fold change was set as greater than 1.5, *p*-value ≤ 0.05). There were 1,002 DEPs identified as significant in a comparison between WRS1/WRL1. There were 1,027 DEPs between WRS1/WRL 2,976 DEPs between WRS2/WRL1, and 911 DEPs between WRS2/WRL2. A total of 193 DEPs, 115 being upregulated and 78 downregulated, were repeatedly identified in the four comparisons (Figure 4B; Supplementary Table S8).

GO enrichment analysis showed that cell and cell parts of the CCs, catalytic activity and binding of the MFs, and cell process and metabolic process of the BPs were the most enriched categories in the proteome of the radicle (Figure 4C). Because proteins usually interact with each other to exert their biological functions, we performed a pathway-enrichment analysis of the DEPs based on the KEGG database (Figure 4D). The DEPs were significantly enriched in phenylpropanoid biosynthesis, photosynthesis, carbon metabolism and genetic information processing. DEPs were also enriched in carbon fixation in photosynthetic organisms, plant-pathogen interaction, starch and sucrose metabolism, mRNA surveillance pathway, and RNA transport. Subcellular localization analysis predicted that the DEPs were mainly located in the chloroplast, cytosol, nucleus and plasma membrane.

### Integration of data from both GWAS and RNA-seq analyses

**Figure 5:**
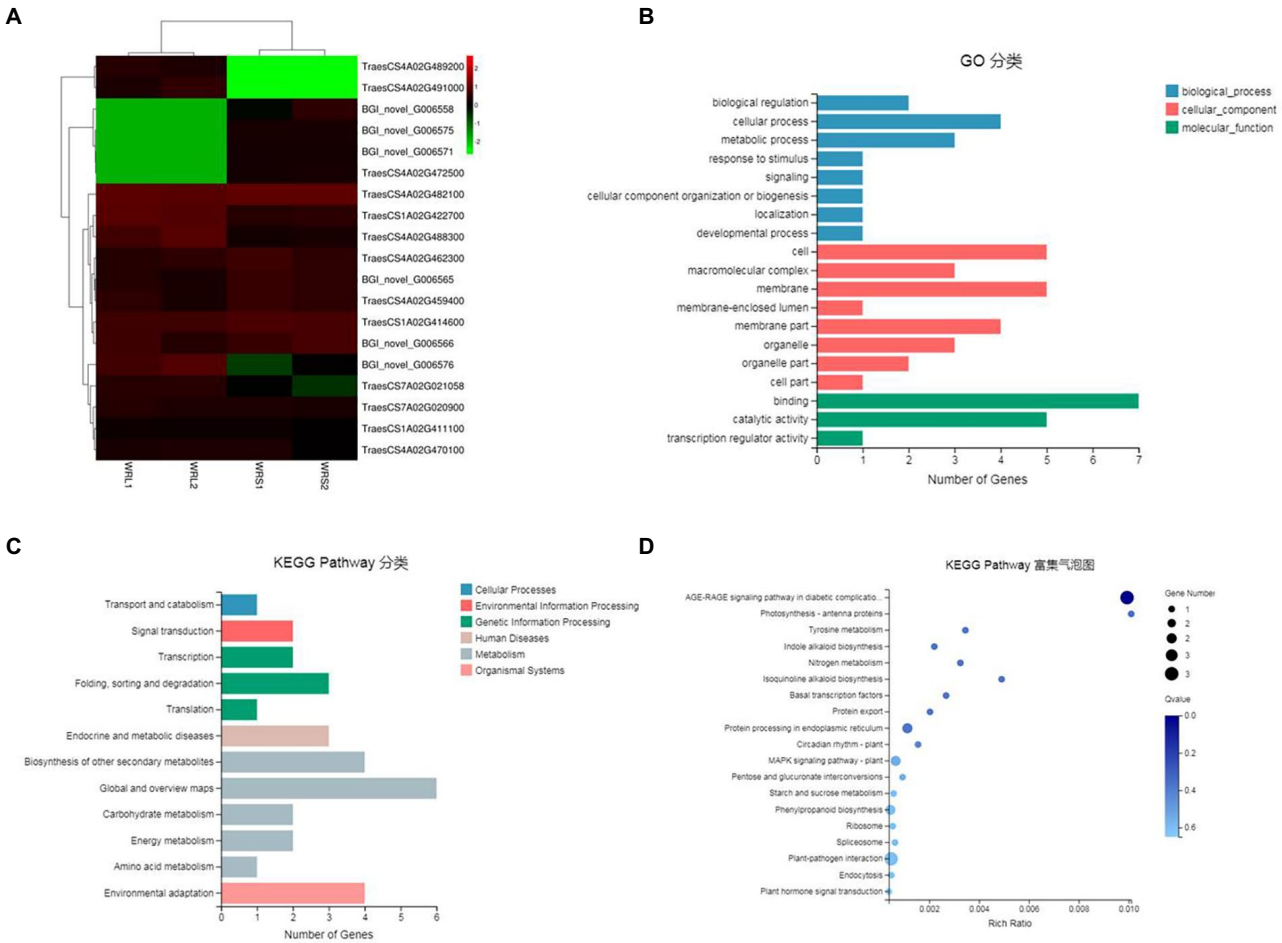
Statistics and annotation of the DEGs in candidate segments of radical length. **(A)** A heat map of 19 candidate genes, **(B)** Candidate genes-enriched GO annotations, **(C)** Candidate genes-enriched KEGG annotations, **(D)** Candidate genes-enriched KEGG annotations.

Among the four stable chromosomal regions of particular interest, a total of 526 genes were retrieved, according to the *Triticum aestivum* reference genome. Combining GWAS and RNA-seq results, 19 candidate genes were selected for their effect on radicle length (Supplementary Table S9). Of these 19 genes, four genes were upregulated in WRL compared to WRS, nine genes were downregulated in WRS VS WRL group, two genes were found to be expressed only in WRL, four genes were expressed only in WRS (Figure 5A). GO enrichment analysis showed that cell and membrane of the CCs, catalytic activity and binding of the MFs, and cell process and metabolic process of the BPs were the most enriched categories in the gene of the radicle (Figure 5B). The KEGG analysis indicated that 19 pathways were enriched, among which, 6 genes were mainly related to global and overview maps, 4 genes were involved in biosynthesis of other secondary metabolites, 4 genes were related to environment adaptation, and 3 genes were participated in endocrine and metabolic diseases (Figure 5C). Candidate genes were mostly enriched in AGE-RAGE signaling pathway in diabetic complications and photosynthesis—antenna proteins, were also enriched in plant-pathogen interaction, MAPK signaling pathway—plant, phenylpropanoid biosynthesis, and protein processing in endoplasmic reticulum (Figure 5D).

### Conjoint analysis of DEPs and DEGs

**Figure 6:**
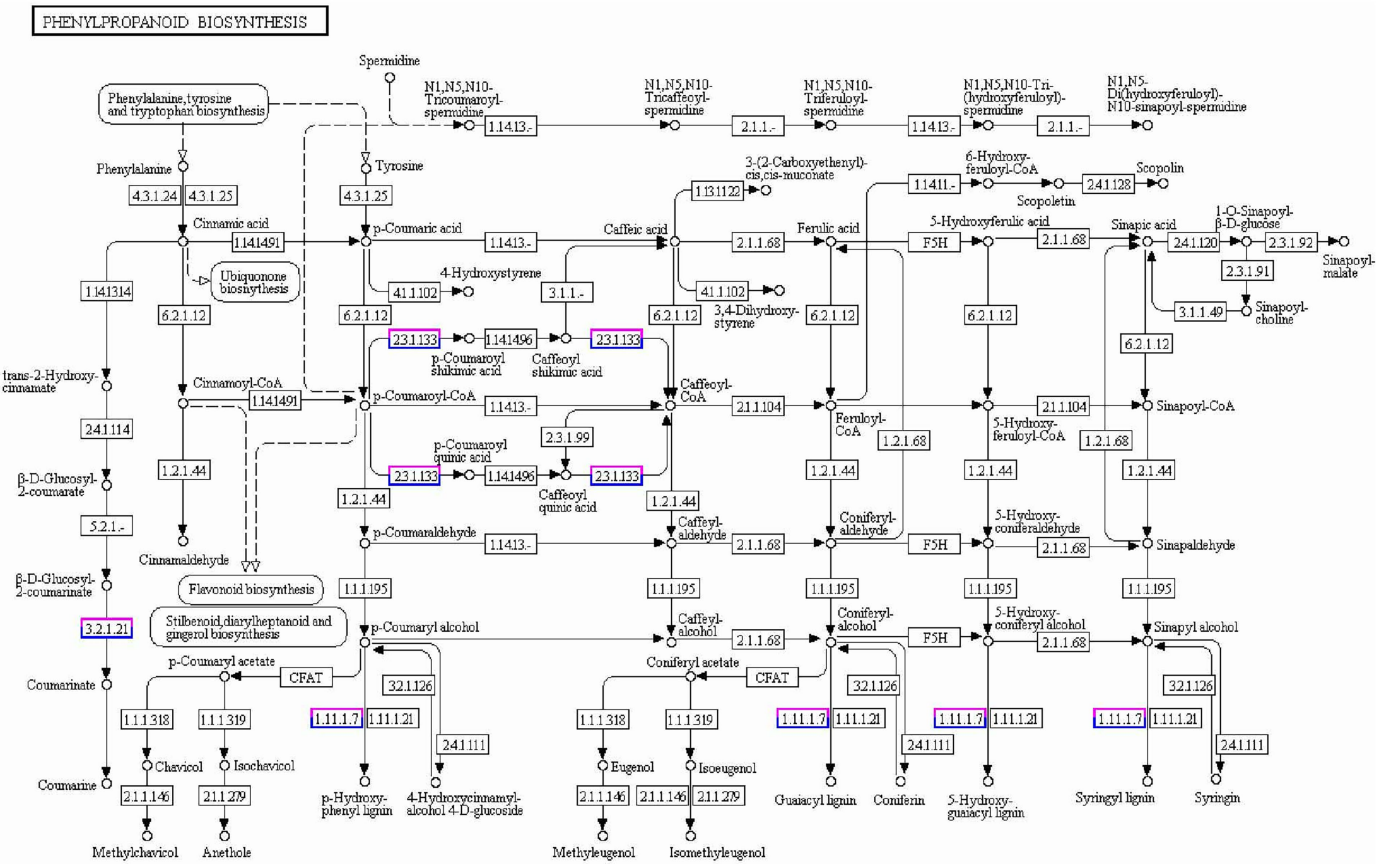
Phenylpropanoid biosynthesis pathways including DEPs and DEGs for wheat radicle length. Red frames are annotated metabolic pathway targets with DEPs, and blue frames are annotated metabolic pathway targets with DEGs.

A conjoint analysis of DEGs and DEPs related to radicle length was performed. The correlation coefficient between the RNA-seq and proteomic data sets was high (0.62), and transcripts were detected for 99.9% of the proteins (Supplementary Figure S5). Based on the quantitative analysis of their expression changes, DEPs and their corresponding genes were used for the conjoint analysis. We observed 19 protein/gene sets that showed the same trend and 5 that showed opposite trends, representing correlation between protein abundance and transcript accumulation in the different accessions. Forty-two pathways were significantly downregulated or upregulated, including photosynthesis, phenylpropanoid biosynthesis, MAPK signaling, indole alkaloid biosynthesis, flavonoid biosynthesis, carbon fixation in photosynthetic organisms, plant-pathogen interaction, and RNA transport (Supplementary Table S10). There were several proteins (12) and genes (103) involved in the key pathways of phenylpropanoid biosynthesis that correlated with radicle length, including *TraesCS1A02G104100/Q42517* and *TraesCS2B02G519100/A9ZPJ6*. In particular, *Q42517*, an N-like peroxidase that catalyzes lignin valorization in the cell wall, was downregulated in WRS compared to WRL (Figure 6). The expression patterns detected by qRT-PCR for these 13 genes were consistent with those in the different profiles, suggesting the reliability and accuracy of the omics data (Supplementary Figure S6).

### Functional domain analysis of *TaCRK6*

Based on these omics analyses, *TraesCS1A02G422700* was one of 19 data points showing the same trend (downregulated in the four comparisons) in both the transcriptome and proteome datasets and was also located in the same region identified by GWAS, 570.52~580.23 Mb region, on chromosome 1A. *TraesCS1A02G422700* was predicted to encode a cysteine-rich receptor-like protein kinase 6 (CRK6) that localizes in the cell membrane. Receptor-like kinases (RLKs) are involved in regulation of plant defense, growth and development. The gene *TraesCS1A02G422700* was designated as *TaCRK6*.

**Figure 7:**
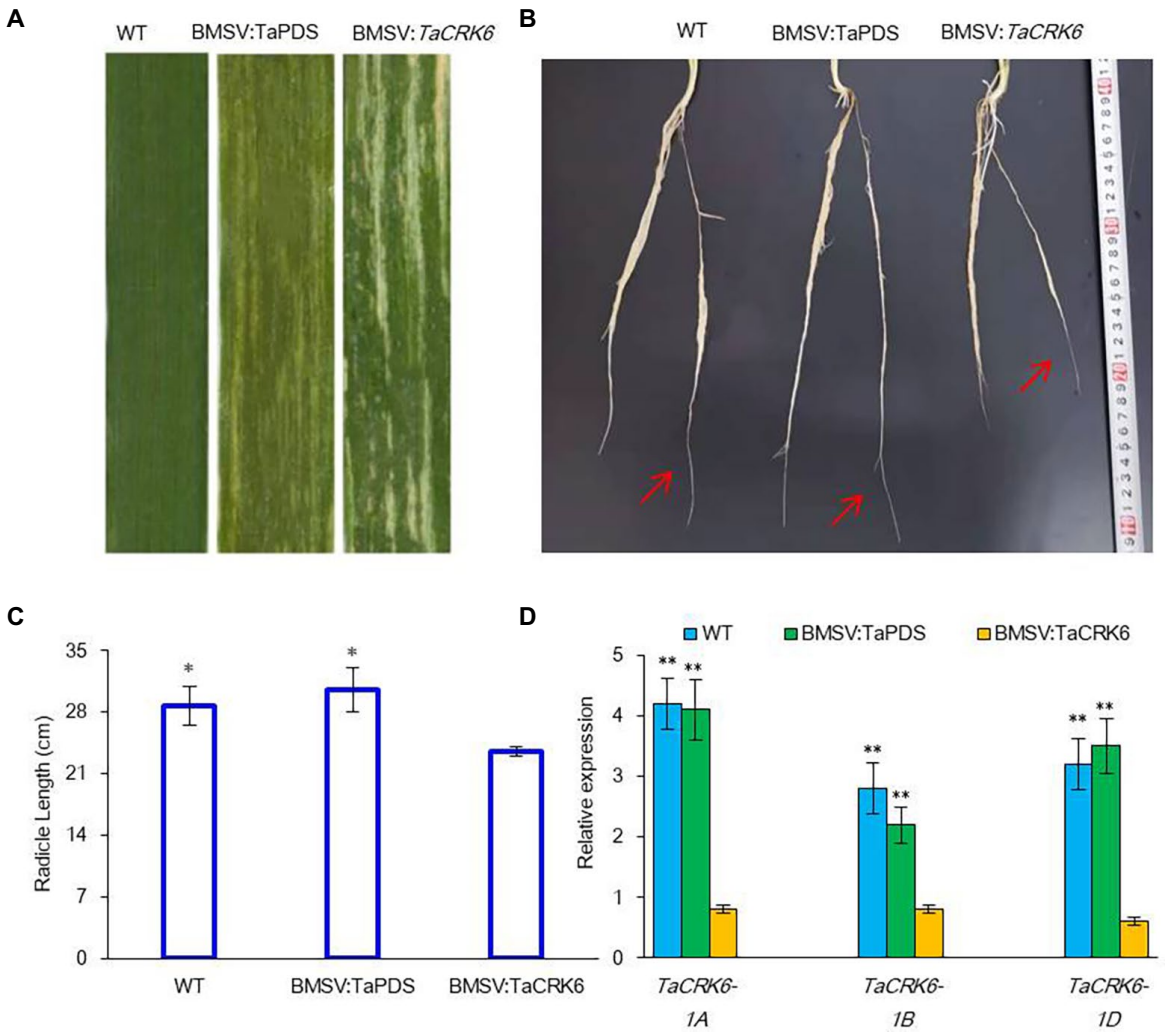
Silencing of *TaCRK6* in wheat using the BSMV-VIGS system. **(A)** BSMV and photobleaching were evident in plants infected by BSMV:PDS after 20 days post-inoculation, **(B)** Radicle length at the third-fourth leaf stage with BSMV:PDS, BSMV:*TaCRK6*, or BSMV:WT at 20 days post-inoculation. Red arrows indicate radicle, **(C)** Average radicle length of different treatments after sowing 30-days, **(D)** Relative transcript levels of *TaCRK6* in knock-down plants and control wheat roots. The values of root are the mean ± SD from four samples, and significant differences are indicated as *p* < 0.05 (*) and *p* < 0.01 (**).

We also explored the function of the *TaCRK6* gene in wheat using the barley stripe mosaic virus-induced gene silencing (BSMV-VIGS) technique. Wheat leaf photobleaching was observed in all plants infected with the BSMV:TaPDS construct at 20 days post-inoculation, suggesting that the endogenous PDS gene was silenced (Figure 7A). After infection with VIGS for 20 d, radicle length of the BSMV:*TaCRK6* accessions were significantly shorter than the WT (wild type) and BSMV:TaPDS accessions (Figures 7B,C). Real-time PCR analysis showed that the transcript level of *TaCRK6* was reduced in the BSMV:*TaCRK6* accessions compared with the WT and BSMV:TaPDS accessions (Figure 7D). These results indicated that TaCRK6 positively improved root development in wheat.

## Discussion

### Morphological differences at the microscopic level in wheat root tips

Root formation and elongation are the result of cell division and differentiation at the root tip. Morphologically, the root tip can be divided into root cap, meristem region, elongation zone and mature region. Cells in the meristem have a higher rate of cell division, cells in the elongation zone have stopped proliferating and began to differentiate, while cells in the mature region are done differentiating and begin to form root hairs and vascular tissue (Caño-Delgado et al., 2010; Mendrinna and Persson, 2015). Through a large-scale screen of 196 wheat accessions, we found that the radicle length varied greatly 10 days after sowing, and that longitudinal morphology of the root tip also differed among the accessions. There was a significant difference in the length of the elongation zone. In the elongation zone of Arabidopsis thaliana, cortical cells are the most sensitive to water (Dietrich et al., 2017), but in maize, cells in the root cap were most sensitive to water while the cells in the elongation zone were sensitive to changes in the gradient of moisture (Wang et al., 2020). In maize under water-stressed conditions, longitudinal extension of the cell wall in the elongation zone was inhibited (Fan et al., 2006). Cell wall formation is controlled by the expression of cell wall remodeling enzymes (Onkokesung et al., 2010), such as H^+^-ATPase (Hager, 2003), xyloglucan endotransglucosylase/hydrolase (XTH) and expansin (Liu et al., 2007; Guo et al., 2009). Enhancing the transcript level of some cell cycle repressors (KRP2, SIM and SHY2) at the meristem–elongation junction affects expression of D-type cyclins, which control cell cycle. Division and differentiation of apical stem cells also regulates root growth (Dewitte et al., 2007; Matsumura et al., 2009; Bishopp et al., 2012; Vaseva et al., 2018). Here we speculate that the difference in length of the radicle was mainly caused by the number of extended cells and cell differentiation in the elongation zone of the root tip.

### Genetic basis of wheat radicle length

Previous studies on root traits of wheat at the seedling stage in greenhouse conditions have identified hundreds of QTLs distributed across all chromosomes. Among the three genomes in hexaploid wheat, 39% of the QTLs were in genome A, 42% in genome B and 19% in genome D, explaining 1.4% to 76.2% of the phenotypic variance (Soriano and Alvaro, 2019). Among them, QTLs for root length were mainly located on chromosomes 1A, 1D, 2B, 5D, 7A, and 7B (Ren et al., 2012; Canè et al., 2014; Cao et al., 2014; Li et al., 2019a,b). However, roots were complicated quantitative traits with lower heritability than shoot traits (Ibrahim et al., 2021; Sharma et al., 2021), and it is hard to distinguish the boundaries of old, new roots and even root tips. The QQ plots for the radicle length in the present study deflated downwards which may be partially attributed to low heritability of the trait, similar results were also earlier reported in case of wheat, rice and barley (Cai et al., 2013; Biscarini et al., 2016; To et al., 2019; Xu et al., 2021) where GWAS was carried out to identify marker trait associations for root length.

In this study, four stable regions, on chromosomes 1A, 4A, and 7A, were associated with radicle length across four hydroponic culture environments. Two of the four regions were on chromosome 4A, and fourteen of the DEGs from the RNA-seq data were located in these regions. The genes *TraesCS4A02G482100* and *TraesCS4A02G470100* are predicted to encode an expansin (A17) and a vegetative cell wall protein (gp1), both of which function in cell wall synthesis and elongation and are upregulated in the long-root accessions. These proteins might disrupt non-covalent bonding between cellulose microfibrils and matrix glucans to cause loosening and expansion of plant cell walls (Voigt et al., 2009; Yu et al., 2011; Lee and Kim, 2013). According to previous results, Xpsr1327_1A (583.36 Mb), which was associated with total root length is approximately 3 Mb from the 570.52~580.23 Mb association region on chromosome 1A (Yang et al., 2014). Excalibur_c8522_1894 (4.89 Mb), which was associated with root number was approximately 3 Mb from the 7.59–10.20 Mb on chromosome 7A (Atkinson et al., 2015). The region might harbor multiple and/or linked genes, as observed in previous studies (Ren et al., 2012; Li et al., 2019a,b). There were five DEGs located in these two regions involved in plant growth and development and plant defense.

### Transcriptome and proteome alterations in wheat radicles of different lengths

The extension of cell walls mainly determines root growth. In maize primary root, polysaccharide cleavage through hydroxyl radical production from H_2_O_2_ directly affects the loosening of the cell wall in the elongation region (Rodríguez et al., 2002; Liszkay et al., 2004). Under water deficit, increased ROS content in root protophores can induce longitudinal extension of the cell wall (Zhu et al., 2008). Combined RNA-seq and proteomic analyses found 3,086 DEGs and 193 DEPs enriched in the same pathways and biological processes in the KEGG/GO analyses, although there was low relativity between the two omics datasets. Some of the DEGs/DEPs revealed significant enrichment for lipid metabolism, especially phenylpropanoid biosynthesis. The lipid synthesis pathway was affected, with laccase and peroxidase as DEPs. Lignin is the major phenolic polymer in plant secondary cell walls, confers rigidity to cell walls, and is at a high level in the cortical and middle column structures, undifferentiated meristem and elongation regions (Zhang et al., 2014; Marcon et al., 2015). Laccase is a multicomponent glycine protein involved in lignin polymerization, and over-expression lignifies plant cells (Liang et al., 2006; Zhao et al., 2013). In the elongation zone of maize primary roots under water stress, lignin content is significantly increased, causing a reduction in elongation of cells (Fan et al., 2006). Here we speculated that the short-rooted accessions, which show higher levels of oxidative enzymes and laccase in root tips, have increased phenylpropanoid biosynthesis and catalysis of lignan-type compounds, thus reducing the extension of cell walls and delaying root elongation.

Some transcription factors (TFs) with significant expression changes at both the mRNA and protein levels during root elongation belong to the MYB, NAC and WRKY families. MYB and NAC transcription factors are known to control lignin metabolism, and MYB transcription factors are regulated by upstream WRKY, bZIP and other transcription factors (Dong and Lin, 2020). Many WRKY factors, which contained C_2_H_2_ zinc finger DNA-binding domains, are not only associated with root development but also plant defense responses (Eulgem and Somssich, 2007). There were eight DEGs/DEPs that significantly enriched plant-pathogen interaction pathways in this study. An increase in the hypersensitive response makes cell walls thinner and more stretchable. The DEGs/DEPs involved in this could include the downregulation of the sugar transport protein 14, the Bowman-Birk type trypsin inhibitor, the putative ribonuclease H protein and the putative xylanase inhibitor, and upregulation of the endoplasmin homolog, deSI-like protein, aspartyl protease family protein. Likewise, a probable WRKY transcription factor 4 that may upregulate defense-related gene expression. The above results show that long and short radicles can perceive the external environment. When stressed, plants can respond to improve their defense mechanisms but at the cost of root growth and function (absorbing nutrients and water).

Ubiquitin-mediated protein is one of the most important enzymes in the protein degradation pathway of eukaryotes (McClellan et al., 2005). Among them, Ubiquitin-specific protease 17 and 21 were differentially phosphorylated by DNA damage, and effected loading of genes associated with DNA double-strand break repair (Kim et al., 2014; Liu et al., 2017). Another a ubiquitin-mediated protease dentin matrix protein (DMP), which takes an active part in remodeling, fusion and fission of the cell membrane during cellular senescence and root development and is expressed in phloem cells and apical cortex cells (Van der Graaff et al., 2006; Kasaras et al., 2012). In the study, some DEGs (*TraesCS2A02G141700, TraesCS2D02G145200,* and *TraesCS2B02G166900*) are predicted to encode DMP. Upregulation of these genes or proteins promotes plant physiological processes, accelerates cell metabolism, stimulates cell division and promotes root growth. There were seven DEGs/DEPs significantly enriched in carbon fixation in photosynthetic organisms, namely seven upregulated invertases (Supplementary Table S10). Invertases catalyze the hydrolysis of sucrose to glucose and fructose, which is irreversible in higher plants (Liu et al., 2013; Ruan, 2014). Increased invertase activity and content may form a powerful carbon metabolism channel that enhances carbohydrate transport from intracellular to extracellular spaces to maintain sugar supply for root growth. This would be accompanied by increased energy metabolism, amino acid synthesis and degradation, storage of nutrients and other metabolic activities, especially in carbon metabolism. Photosynthesis and ABC transporters were enhanced in plants with faster elongation of root, which would result in more carbohydrate distribution in underground parts and provide raw materials for cell division and elongation.

**Figure 8:**
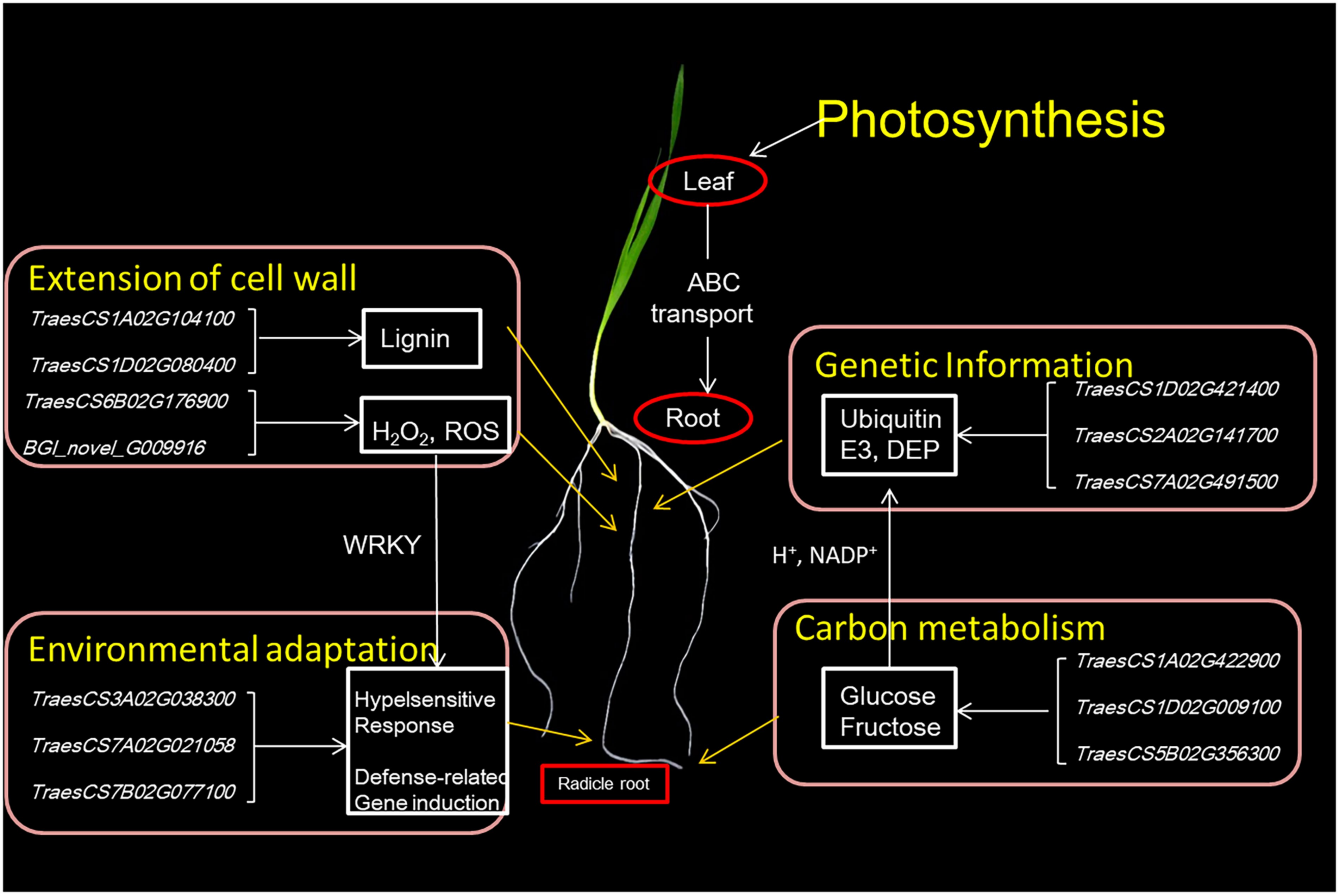
A molecular regulatory model controlling wheat radicle length.

From these multiple results, we propose a model for the length of radicle in wheat (Figure 8). Carbohydrate is transported from photosynthesizing source leaves to the radicle. There was a significant number of DEGs targeting different DEPs that play important roles in regulating carbohydrate metabolism, energy metabolism, and synthesis and degradation of DNA and RNA, which would all work together to ensure the supply of raw materials for cell division needed to maintain division and elongation of the cells. At the same time, proteins needed for the synthesis of lignin and peroxide (H_2_O_2_, ROS) were upregulated.

### Prediction of candidate gene involved in wheat radicle length

Because candidate loci/genes for quantitative traits from a single omic result only contribute small genetic effects, especially for crops with large genomes, the expression of a gene/protein can greatly vary among different omics. Yao et al. (2020) showed that more than one-fourth of the potential resistance genes detected in a GWAS were differentially expressed in the transcriptome analysis for maize resistance to ear rot. Previous studies have also revealed the detailed functions of candidate genes and proteins specifically expressed in roots of different species by combined protein-transcript analyses (Wang et al., 2019). Combining GWAS and RNA-seq results, 19 DEGs were localized to four stable chromosomal regions in relation to radicle length. Among them, *TaCRK6* was also upregulated in WRL-*vs*-WRS at both the transcript and protein levels. There are two homologous for the gene encoding T*aCRK6*, namely *TraesCS1B01G454000* and *TraesCS1D02G430900* in wheat. Quantitative analysis of these three homologous genes found that only *TaCRK6* was differentially expressed between the long and short roots (Supplementary Figure S5). This suggested that *TaCRK6* plays a major role in regulating the length of the wheat radicle.

**Figure 9:**
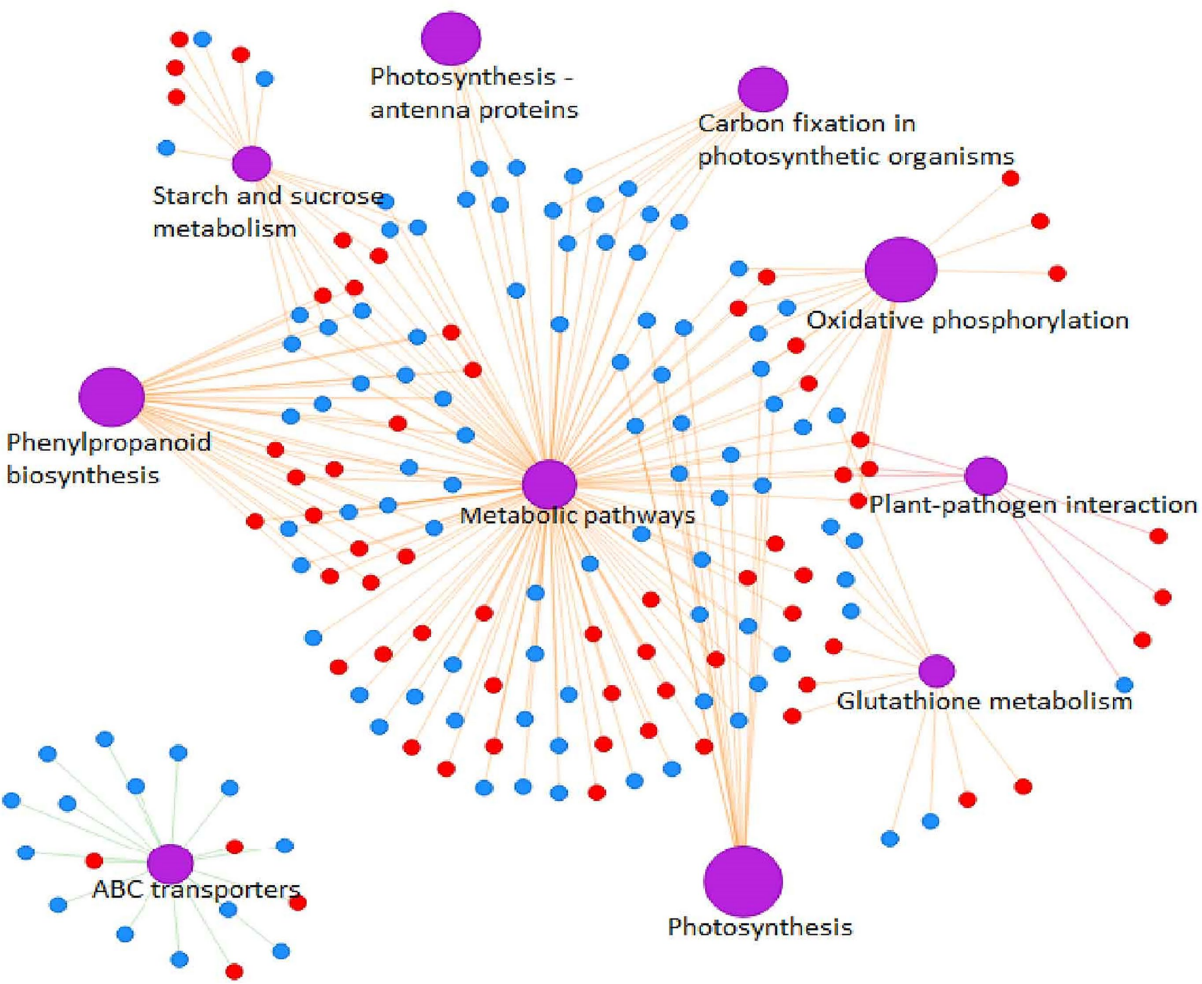
Protein–protein interaction (PPI) network analysis of the DEPs between long and short wheat radicles. Red circles represent upregulated proteins and blue circles downregulated proteins. Edges with purple represent classes of KEGG pathway.

*TraesCS1A02G422700* encodes a cysteine-rich receptor-like protein kinase 6 (CRK6), which is a member of the largest subfamily of receptor-like protein kinases (RLK). The extracellular domain of CRK is predicted to contain two named Domain of Unknown Function 26 (DUF 26) motifs, a disulfide bond within the motif is involved in formation of three-dimensional structures of protein and is controlled by reactive oxygen species. In wheat, expression of CRKs in plasma membrane and epidermal cells increased significantly by infection with *Rhizoctonia cerealis* or application of exogenous ABA (Yang et al., 2013). Some CRK genes are involved in multiple biological pathways in *Arabidopsis thaliana*. For instance, *AtCRK5* is involved in regulation of plant growth and development (Burdiak et al., 2015), *AtCRK6* and *AtCRK7* are involved in signal transduction of extracellular oxidative stress (Idanheimo et al., 2014), *AtCRKl7* and *AtCRKl8* respond to SA and ABA signal transduction, respectively (Xin et al., 2005). Kinases that contain leucine-rich repeat (LRR) domains, which forms a *β-*sheet among the most conserved amino acids in the repeating unit, are involved in the interaction among proteins to control the occurrence of somatic embryos (DeYoung and Innes, 2006), the formation of apical meristem and lateral root primordium (Suzaki et al., 2004), and the arrangement and differentiation of root epidermal cells (Becraft et al., 2001; Kwak et al., 2005). This family of proteins plays a crucial role in sensing external stimuli, activating downstream signaling pathways, regulating responses to pathogen infection, and regulating growth and development. A protein–protein interaction network for all common DEPs showed that *TaCRK6* might regulate the synthesis of phenylpropanoid metabolites through a series of complex metabolic networks (Figure 9). The BSMV-mediated gene silencing of *TaCRK6* also showed that *TaCRK6* was significantly shortened radicle length. Therefore, *TaCRK6* located on chromosome 1A might be a major gene controlling wheat radicle length.

### Conclusion

Differences in the length of wheat radicle are mainly caused by differences in the length of the elongation zone at the root tip. A comparison of the transcripts and proteins in extremely long and extremely short roots found DEGs/DEPs related to cell wall synthesis, carbon metabolism, transmission of genetic information and plant defense. A combination of data from RNA-seq and iTRAQ showed that phenylpropanoid biosynthesis was active, regulating lignin synthesis and plant defense, which then affected the extension of the cell wall in the apical elongation region. Four stable chromosomal regions were linked to wheat radicle length through a GWAS and were detected on chromosomes 1A, 4A, and 7A. A combination of GWAS and RNA-seq analyses revealed that the expression patterns of 19 genes changed among the four accessions. Among these genes, TaCRK6 was upregulated in both the iTRAQ and qRT-PCR data. VIGS-induced gene silencing analyses revealed that TaCRK6 could control radicle length of wheat. TaCRK6 is a candidate gene involved in the determination of wheat radicle length.

## Data availability statement

The data presented in the study are deposited in the NCBI and Proteome Xchange Consortium repository, accession numbers PRJNA861912 and IPX0004728000, respectively.

## Author contributions

FX, XY, XZ and DH conceived the topic. KZ and SC provided gene chip and wheat material, XY, SC, XZ and SZ helped to draft the manuscript. FX, SC, CY and XY performed the phenotypic evaluation and helped with data analysis. FX supervised the whole study and provided assistance for manuscript preparation. All authors read and approved the manuscript.

## Funding

The work was supported by the National Key Research and Development Program of China “Science and Technology Innovation of High Grain Production Efficiency” (2018YFD0300701) and National Key Basic Research Program of China “Interaction Network of quality and yield traits and whole genome selection model in wheat” (2014CB138105).

## Conflict of interest

The authors declare that the research was conducted in the absence of any commercial or financial relationships that could be construed as a potential conflict of interest.

## Publisher’s note

All claims expressed in this article are solely those of the authors and do not necessarily represent those of their affiliated organizations, or those of the publisher, the editors and the reviewers. Any product that may be evaluated in this article, or claim that may be made by its manufacturer, is not guaranteed or endorsed by the publisher.

## Footnotes

^1^ http://wheat.pw.usda.gov/ggpages/topics/Wheat_660_SNP_array_developed_by_CAAS.pdf

^2^ https://github.com/YinLiLin/R-CMplot

^3^ https://github.com/BGI-flexlab/SOAPpnuke

^4^ ftp://ftp.ensemblgenomes.org/pub/plants/release-32/fasta/triticum_aestivum/

^5^ https://bowtie-bio.sourceforge.net/Bowtie2/index.shtml

^6^ https://geneontology.org/

^7^ https://www.kegg.jp/

^8^ https://wolfpsort.hgc.jp/

^9^ https://string-db.org/

^10^ https://bioinfogp.cnb.csic.es/tools/venny/index.html

## Supplementary material

SUPPLEMENTARY FIGURE S1 Quantile-quantile plots for radicle length across 196 accessions with six models.

SUPPLEMENTARY FIGURE S2 DEG-enriched GO annotations.

SUPPLEMENTARY FIGURE S3 DEG-enriched KEGG annotations.

SUPPLEMENTARY FIGURE S4 **(A)** Enriched KEGG annotations specifically expressed in WRL; **(B)** KEGG annotations specifically expressed in WRS.

SUPPLEMENTARY FIGURE S5 Correlation coefficient between RNA-seq and proteomic datasets.

SUPPLEMENTARY FIGURE S6 The mRNA and gene/protein abundance changes of 14 selected genes in the study.
