# Supplementary figures and images for: Genome-wide association, RNA-seq and iTRAQ analyses identify candidate genes controlling radicle length of wheat
Source: Front Plant Sci. 2022 Sep 28;13:939544. doi: 10.3389/fpls.2022.939544 (PMC9554269; doi:10.3389/fpls.2022.939544)

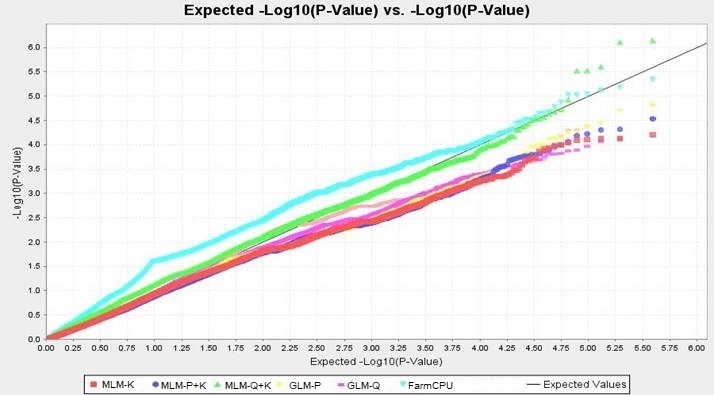

Supplement: SUPPLEMENTARY FIGURE S1 — Quantile-quantile plots for radicle length across 196 accessions with six models. [file Image_1.JPEG]

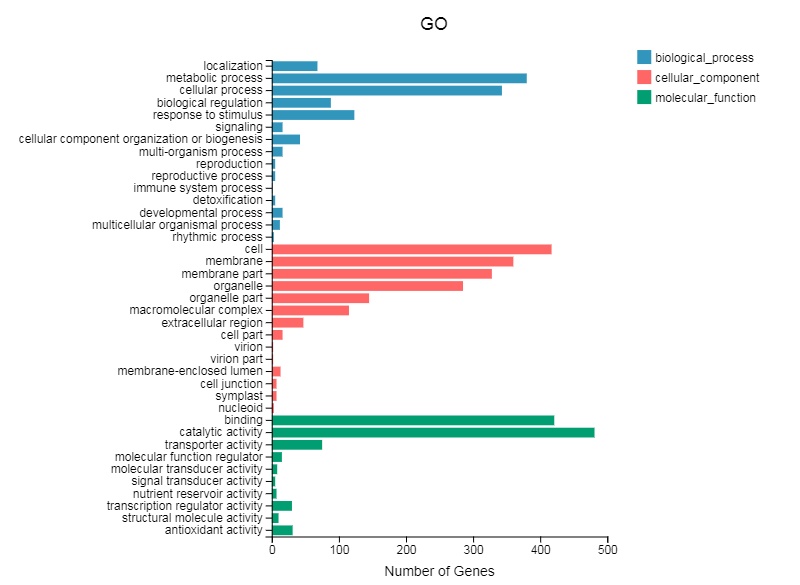

Supplement: SUPPLEMENTARY FIGURE S2 — DEG-enriched GO annotations. [file Image_2.PNG]

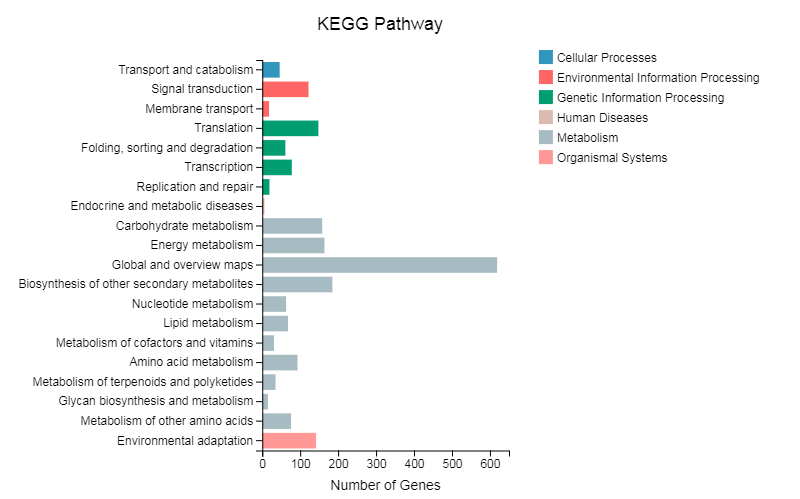

Supplement: SUPPLEMENTARY FIGURE S3 — DEG-enriched KEGG annotations. [file Image_3.PNG]

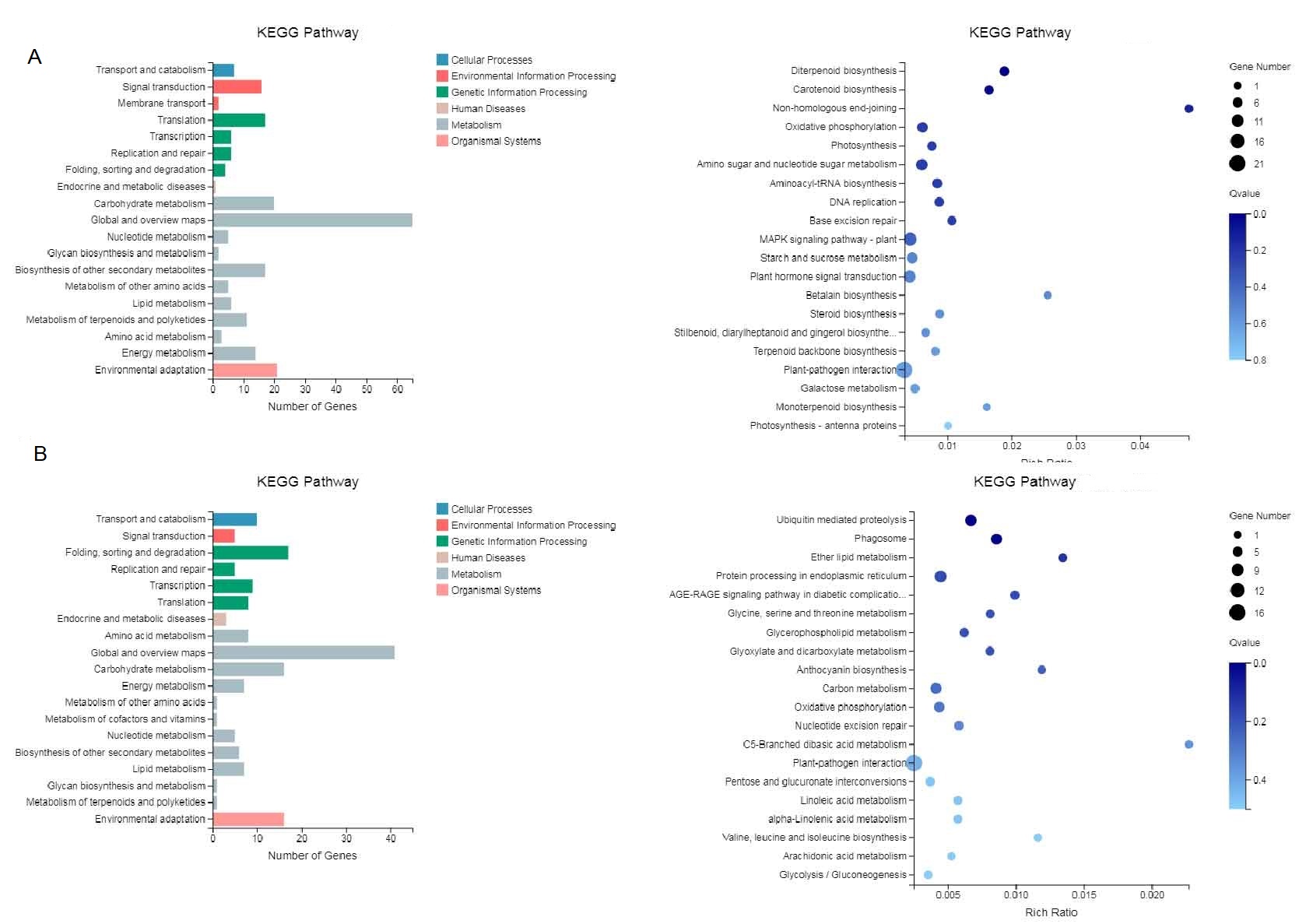

Supplement: SUPPLEMENTARY FIGURE S4 — (A) Enriched KEGG annotations specifically expressed in WRL; (B) KEGG annotations specifically expressed in WRS. [file Image_4.JPEG]

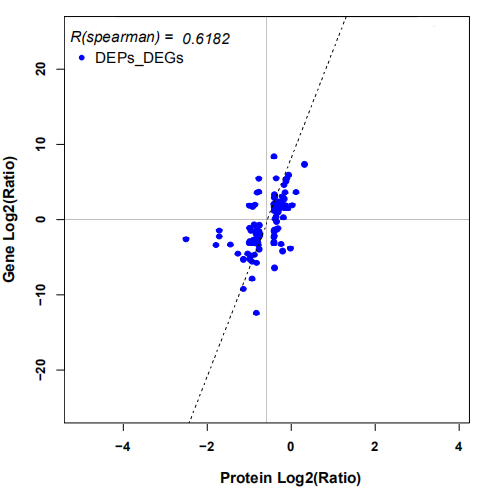

Supplement: SUPPLEMENTARY FIGURE S5 — Correlation coefficient between RNA-seq and proteomic datasets. [file Image_5.PNG]

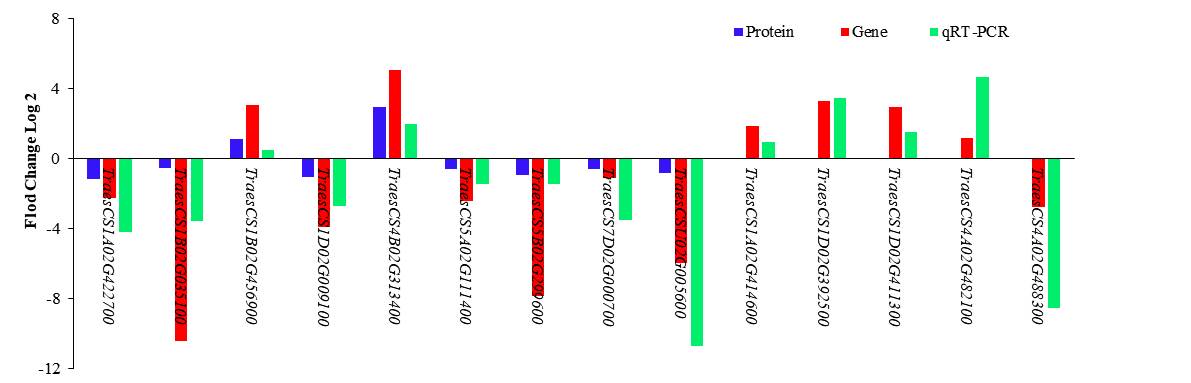

Supplement: SUPPLEMENTARY FIGURE S6 — The mRNA and gene/protein abundance changes of 14 selected genes in the study. [file Image_6.JPEG]
